# A cross-sectional analysis of the association between screen-based sedentary behavior and erectile dysfunction in US adult males

**DOI:** 10.1038/s41598-025-02976-y

**Published:** 2025-05-24

**Authors:** Xiaoxiao Wang, Zhenqiang He, Chongzhou Liao, Pu Guo, Yue Zhao, Wei Xiong

**Affiliations:** 1 Department of Organ Transplantation, School of Medicine, Sichuan Provincial People’s Hospital, University of Electronic Science and Technology of China, Chengdu, China; 2 School of Medical and Life Sciences, Chengdu University of Traditional Chinese Medicine, Chengdu, China; 3 School of Medicine, University of Electronic Science and Technology of China, Chengdu, China; 4 Department of Radiation Oncology, Radiation Oncology Key Laboratory of Sichuan Province, Sichuan Clinical Research Center for Cancer, Sichuan Cancer Hospital & Institute, Sichuan Cancer Center, Affiliated Cancer Hospital of University of Electronic Science and Technology of China, Chengdu, China; 5 Department of Urology, School of Medicine, Sichuan Provincial People’s Hospital, University of Electronic Science and Technology of China, Chengdu, China

**Keywords:** Erectile dysfunction, Screen-based sedentary behavior, NHANES, Cross-sectional studies, Health care, Urogenital diseases

## Abstract

Despite previous studies, the association between screen-based sedentary behavior (SB) and erectile dysfunction (ED) remains unclear. This study aimed to investigate the relationship between screen-based SB and ED using data from the 2001–2004 National Health and Nutrition Examination Survey (NHANES). A total of 4,047 participants were included, of whom 1,192 (29.5%) had ED. Multivariable logistic regression models were used to analyze this association. After adjusting for potential confounders, screen-based SB exceeding 2 h per day was significantly associated with an increased risk of ED (OR = 1.32; 95% CI, 1.12–1.56; *p* < 0.0001). Subgroup analysis further confirmed a consistent and independent positive association across different population groups (all p for interaction > 0.05). These findings suggest a significant relationship between prolonged screen-based SB and ED in U.S. adult males. Further prospective studies are needed to confirm these results.

## Introduction

Erectile dysfunction (ED), defined as the inability to achieve or sustain an erection sufficient for satisfactory sexual performance, is a condition that generally escalates with age^1^. The prevalence is under 10% for individuals below 50, but it increases to 20-40% for those aged 60–69, and can range from 50% to even 100% in individuals over 70^2–5^. ED can be categorized as psychogenic, organic (including neurogenic, hormonal, arterial, penile cavernosal, or drug-induced), or a blend of both^6^. Age and diabetes are recognized as the primary risk factors for ED^7^. Moreover, associations have been established between ED and behaviors such as smoking, alcohol or drug abuse, sleep disorders, obesity, and metabolic syndromes^8–11^. Although ED is not life-threatening, it is often considered as an early warning sign for cardiovascular disease (CVD), due to the shared risk factors such as hypertension, obesity, and diabetes, as well as pathophysiological links including endothelial dysfunction, chronic inflammation, aging, and low plasma testosterone levels^12^ Recent clinical guidelines recognize ED as an important indicator for assessing cardiovascular risk^13,14^ Early identification and treatment of ED may provide valuable opportunities for the prevention of CVD and reduction of mortality, highlighting its significance in both research and clinical practice.

Sedentary behavior (SB) is defined as any waking activity that requires an energy expenditure of less than 1.5 METs while in a sitting or reclining posture^15,16^. Television viewing, a common and widespread form of screen-based SB, is prevalent, with a recent cross-sectional study from the American Time Use Survey revealing that 60% of Americans engage in at least two hours of daily television watching. The correlation between ED and lifestyle has been a subject of extensive research over the past few decades, yielding inconsistent results. Furukawa et al. discovered a potential positive correlation between self-reported sitting time and ED in Japanese individuals diagnosed with type 2 diabetes mellitus^17^. However, contradictory findings have been reported elsewhere^18^. Hence, there is an urgent need for larger studies involving a more general population.

We propose a potential correlation between screen-based SB and ED. The objective of this study is to elucidate the relationship between screen-based SB and ED by leveraging a substantial database, incorporating comprehensive demographic data, and mitigating the effects of confounding variables.

## Materials and methods

### Patient selection

The National Health and Nutrition Examination Survey (NHANES) is a cross-sectional, population-based study designed to collect data on the health and nutritional status of households in the United States. The project annually surveys approximately 5,000 individuals, representing a national sample, from counties nationwide. The interview component of NHANES encompasses demographic, socioeconomic, nutritional, and health-related topics. The physical examination includes physiological evaluations and laboratory tests. The National Heart, Lung, and Blood Institute (NHLBI) Research Ethics Review Board approved NHANES and all participants provided informed consent before initiation of the study^19^.

**Fig. 1:**
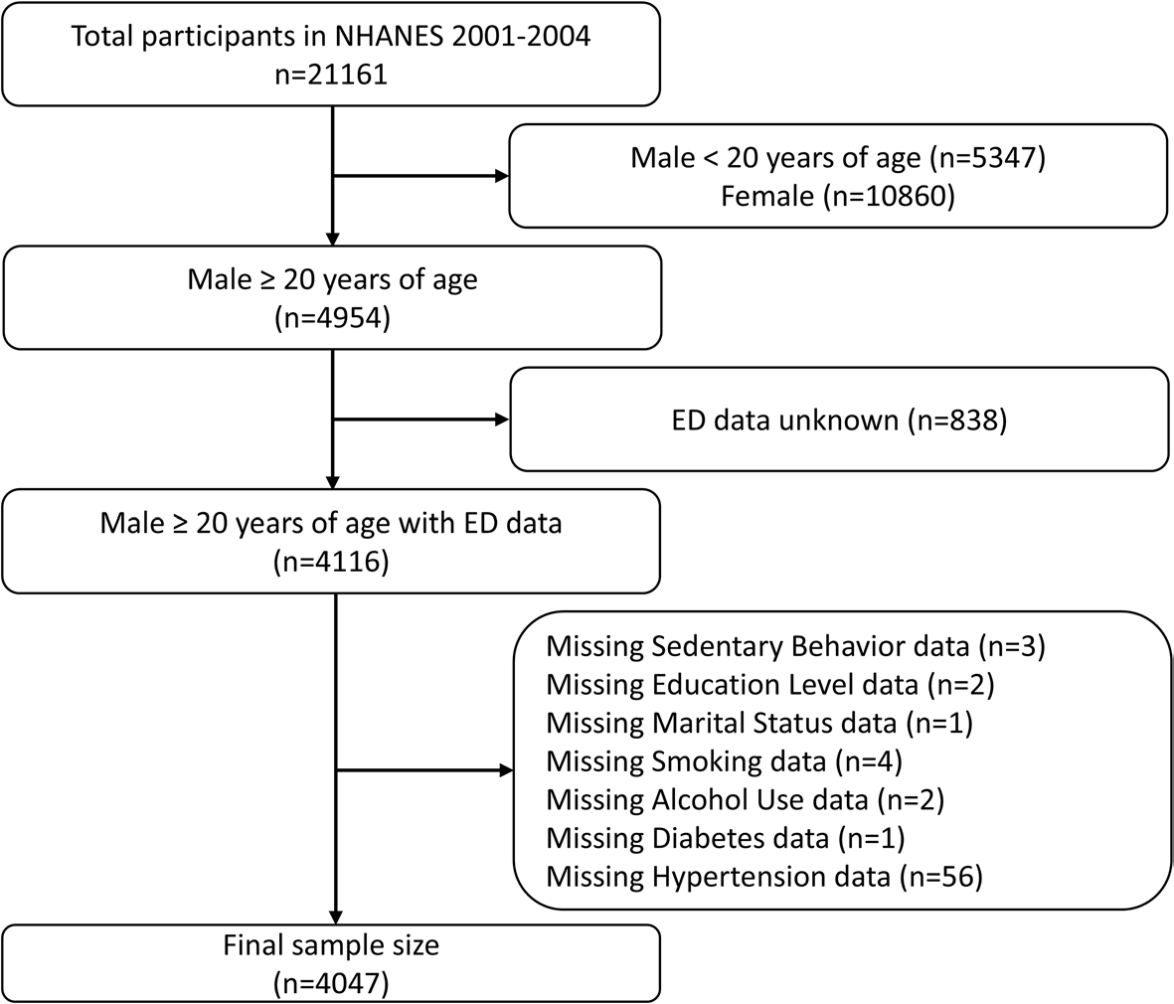
Flow chart of the sample selection process.

This cross-sectional study analyzed data derived from two survey cycles, 2001–2002 and 2003–2004, as the NHANES workgroup only administered the ED questionnaire during this period. The study initially included a total of 21,161 participants. However, following exclusion criteria were applied: (1) Females (*n* = 10860), (2) Males under 20 years of age (*n* = 5347), (3) Unknown ED data (*n* = 838), (4) Missing data on screen-based SB (*n* = 3), (5) Missing data on Education Level (*n* = 2), (6) Missing data on Marital Status (*n* = 1), (7) Missing data on Smoking (*n* = 4), (8) Missing data on Alcohol Use (*n* = 2), (9) Missing data on Diabetes (*n* = 1), and (10) Missing data on Hypertension (*n* = 56). Consequently, a final cohort of 4047 cases was utilized for the study. (Fig. 1)

### Assessment of screen-based SB

The Physical Activity Questionnaire was employed to evaluate screen-based SB. During the 2001–2002 cycle, the pertinent variable was labeled PAD480, and individuals aged 16 and over were asked, “In the past 30 days, how much time, on average, did you generally spend sitting around watching TV, videos, or using a computer on a daily basis, outside of work?” In the 2003–2004 cycle, the variable was renamed to PAD590, and the question was revised to, “Over the past 30 days, on average about how many hours per day did you sit and watch TV or videos?” for individuals aged 2 and over. The response options for both questions were identical, including none, less than 1 h, 1 h, 2 h, 3 h, 4 h, or 5 h or more per day. Based on the similarities in their measurement, particularly in the response options, the two variables were amalgamated into one to evaluate screen-based SB. Drawing on previous studies^20–24^, we further classified the duration of watching television or videos into two categories: less than 2 h per day and 2 or more hours per day. These cutoffs also approximated the median values in the present study population.

### Assessment of ED

Measurement of ED in the present study was conducted by a single question self-assessment in the Massachusetts Male Aging Study (MMAS)^25^. We chose the assessment method based on its widespread use and validation in large epidemiological studies^26^. Participants’ ability to sustain an erection was evaluated through the question, “Many men experience problems with sexual intercourse. How would you describe your ability to achieve and maintain an erection suitable for satisfactory intercourse?” The response options included “always or almost always able”, “usually able”, “sometimes able”, and “never able”. In this study, participants who reported being “sometimes able” or “never able” to maintain an erection were classified as having ED. Conversely, those who stated they were “usually able” or “always or almost always able” to maintain an erection were categorized as not having ED.

### Covariates of interest

In this study, covariates encompassed age, race, educational attainment, marital status, family poverty income ratio (PIR), Body Mass Index (BMI), diabetes, hypertension, smoking status, and alcohol consumption. Participants were stratified as poor (PIR ≤ 1.3), near poor (1.3 < PIR < 3.5), and non-poor (PIR ≥ 3.5). BMI was categorized into normal (< 25), overweight (25.0–30.0), and obese (≥ 30.0). The presence of diabetes was determined by a history of physician-diagnosed diabetes. Participants were considered hypertensive if they had been diagnosed with high blood pressure by a physician. The smoking status was affirmed if participants responded “yes” to having smoked at least 100 cigarettes in their lifetime. Male participants who affirmed having consumed 12 alcoholic drinks in any one year were classified as drinkers.

### Statistical analysis

The baseline characteristics of the study population were statistically delineated by ED and non-ED subgroups. Categorical variables are presented as weighted survey proportions accompanied by 95% confidence intervals. Chi-square tests were employed to evaluate differences in categorical variables between participants with and without ED. Multivariate logistic regression analyses were conducted to examine the association between screen-based SB and ED, with the calculation of 95% confidence intervals (CI) and odds ratios (OR). The research utilized three models: In the crude model, no confounding factors were adjusted. In Model 1, age and race were adjusted; in Model 2, age, race, body mass index, marital status, education level, poverty income ratio, smoking status, and alcohol consumption status were adjusted. Additionally, subgroup analyses were performed to investigate the relationship between ED and screen-based SB within different subgroups. A P-value of less than 0.05 was considered statistically significant. Data analysis was performed using Empower Stats software (versions 2.0 and 4.2).

## Results

### Baseline characteristics

The study comprised a total of 4047 participants with a mean age of 50.3 ± 18.7 years, among whom 1192 (29.5%) reported a history of ED. We applied the 2-year interview weights (WTINT2YR) for the 2001–2004 cycles, as our key variables of interest were derived from survey questionnaires administered during household interviews. For pooled analyses across multiple survey cycles (2001–2002 and 2003–2004), weights were recalculated following National Center for Health Statistics (NCHS) guidelines by dividing the WTINT2YR by 2 to account for combined cycles. Our final analysis sample represents an estimated population of 97.6 million US adult males. Table 1 presents the baseline clinical and demographic characteristics of the study population. Notable statistical differences were observed between the ED and non-ED groups across variables such as age, education level, marital status, PIR, BMI, hypertension, diabetes, alcohol consumption, smoking, and screen-based SB (all *p* < 0.001). Additionally, a significant increase in ED prevalence was observed among participants with sedentary time exceeding 2 h.

**Table 1:** Baseline characteristics of participants by a history of erectile dysfunction, weighted.

Characteristic	History of erectile dysfunction		*P*-value
	No (*n* = 2855)	Yes (*n* = 1192)	
Age, years (%)			< 0.0001
< 50	73.8(71.5,76.1)	19.4 (16.7,22.4)	
≥ 50	26.2(24.1,28.5)	80.6(77.6,83.3)	
Race (%)			0.2238
Mexican American	7.7(5.9,10.0)	6.6(4.1,10.5)	
Other Hispanic	4.0(2.6,6.2)	5.2(2.3,11.5)	
Non-Hispanic White	74.0(69.9,77.7)	76.5 (70.4,81.7)	
Non-Hispanic Black	10.3(8.2,12.8)	8.6(6.4,11.5)	
Other Race	4.1(3.1,5.3)	3.1 (2.0,4.7)	
Education Level (%)			< 0.0001
Less than high school	13.8(12.4,15.4)	28.7 (24.7,33.1)	
High school	27.9(25.8,30.2)	23.4 (20.5,26.6)	
Above high school	58.2(55.7,60.7)	47.9(43.9,51.8)	
Marital status (%)			< 0.0001
Married or living with partner	68.1(65.3,70.7)	76.2(73.2,79.0)	
Living alone	31.9(29.3,34.7)	23.8(21.1,26.8)	
Family PIR (%)			< 0.001
< 1.3	15.1(13.3,17.1)	18.8(15.0,23.1)	
≥ 1.3and < 3.5	37.2(34.8,39.8)	44.8(40.9,48.7)	
≥ 3.5	47.7(44.6,50.8)	36.5(32.4,40.9)	
BMI (kg/m^2^) (%)			0.0005
< 25	30.5(28.5,32.6)	23.3(20.7,26.7)	
≥ 25and < 30	41.7(39.2,44.1)	43.1(39.7,46.5)	
≥ 30	27.8(25.7,30.1)	33.6(29.8,37.6)	
Hypertension (%)			< 0.001
Yes	21.3(19.0,23.9)	50.0 (47.1,53.0)	
No	78.7(76.1,81.1)	50.0 (47.1,53.0)	
Diabetes (%)			< 0.001
Yes	3.9(3.1,5.0)	21.5(18.2,25.3)	
No	95.1(94.2,95.9)	76.9(72.9,80.5)	
Borderline	1.0(0.6,1.5)	1.6(0.8,3.1)	
Alcohol Use (%)			0.0014
Yes	84.3(79.9,87.8)	79.3(74.9,83.1)	
No	15.8(12.2,20.1)	20.7(16.9,25.1)	
Smoking (%)			< 0.001
Yes	54.6(51.6,57.5)	69.2(65.7,72.6)	
No	45.4(42.5,48.4)	30.8(27.4,34.3)	
Sedentary Behavior (%)			< 0.0001
≤ 2 h/d	61.7(58.8,64.5)	45.3(42.0,48.6)	
> 2 h/d	38.3(35.5,41.2)	54.7(51.4,58.0)	

### The association between screen-based SB and ED

The association between screen-based SB and ED was examined in three models: crude, minimally adjusted, and fully adjusted. This was achieved using weighted multivariable logistic regression. As displayed in Table 2, a significant positive correlation was found between screen-based SB and ED across all models.

**Table 2:** Multivariable logistic regression for the association between screen-based sedentary behavior (binary categorical) and erectile dysfunction.

Exposure	Crude Model		Model1^a^		Model2^b^	
	OR (95% CI)	*P*-value	OR (95% CI)	*P*-value	OR (95% CI)	*P*-value
SB ≤ 2 h	Reference		Reference		Reference	
SB > 2 h	1.72(1.51,1.98)	< 0.001	1.51(1.29,1.77)	< 0.001	1.32(1.12,1.56)	0.001

OR, odds ratio; 95% CI, 95% confidence interval.

Crude Model: Unadjusted; Model 1^a^: Adjusted for age and race; Model 2^b^: Adjusted for age, race, education level, marital status, the family poverty income ratio (PIR), body mass index (BMI), hypertension, diabetes, alcohol intaking, and smoking status.

The results indicated that screen-based SB was linked to an increased likelihood of ED. This association was significant in both our crude model (OR = 1.72; 95% CI, 1.51–1.98, *p* < 0.001) and minimally adjusted model (OR = 1.51; 95% CI, 1.29–1.77, *p* < 0.001). In the fully adjusted model, a positive correlation persisted between sitting time exceeding 2 h per day and ED (OR = 1.32; 95% CI, 1.12–1.56, *p* < 0.0001). To validate the cut-off point of 2 h, we further analyzed all screen-based time reported in the questionnaire using logistic regression. In the fully adjusted model, durations of no TV watching, 1 h, and 2 h were not statistically significant, whereas durations of 3 h (OR = 1.60, 95%CI: 1.12–2.29), 4 h (OR = 1.51, 95%CI: 1.02–2.24), and 5 or more hours (OR = 1.49, 95%CI: 1.03–2.14) were significantly associated with ED (Table 3).

**Table 3:** Multivariable logistic regression for the association between screen-based sedentary behavior (multivariate categorical) and erectile dysfunction.

Exposure	Crude Model		Model1^a^		Model2^b^	
	OR (95% CI)	*P*-value	OR (95% CI)	*P*-value	OR (95% CI)	*P*-value
SB < 1 h	Reference		Reference		Reference	
SB = 1 h	1.15(0.84,1.56)	0.3841	1.24(0.86,1.80)	0.2512	1.22(0.83,1.79)	0.3105
SB = 2 h	1.47(1.12,1.93)	0.0053	1.38(0.99,1.92)	0.0548	1.35(0.96,1.90)	0.0840
SB = 3 h	2.09(1.58,2.78)	< 0.0001	1.73(1.23,2.45)	0.0018	1.60(1.12,2.29)	0.0095
SB = 4 h	2.08(1.53,2.84)	< 0.0001	1.67(1.14,2.43)	0.0082	1.51(1.02,2.24)	0.0396
SB ≥ 5 h	2.44(1.84,3.24)	< 0.0001	1.98(1.40,2.80)	0.0001	1.49(1.03,2.14)	0.0321
None	1.49(0.76,2.94)	0.2500	1.86(0.80,4.32)	0.1480	1.59(0.67,3.79)	0.2902

OR, odds ratio; 95% CI, 95% confidence interval.

Crude Model: Unadjusted; Model 1^a^: Adjusted for age and race; Model 2^b^: Adjusted for age, race, education level, marital status, the family poverty income ratio, body mass index, hypertension, diabetes, alcohol intake, and smoking status.

### Subgroup analysis

**Fig. 2:**
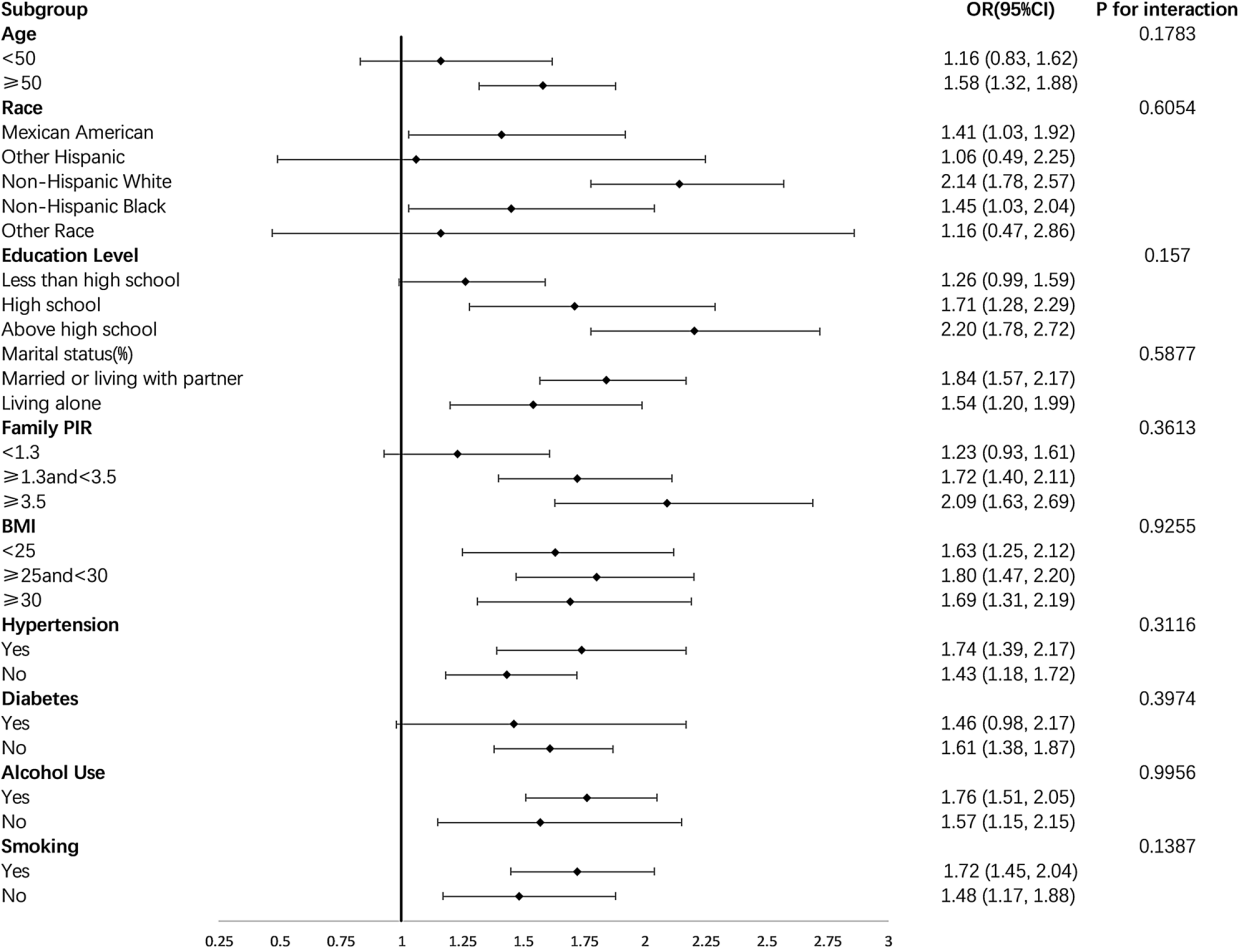
Subgroup analysis for the relationship between screen-based sedentary behavior and erectile dysfunction. All factors including age, race, education level, the family poverty income ratio (PIR), body mass index (BMI), hypertension, diabetes, alcohol intake, and smoking status had no impact on the independent positive association between SB and ED. All subgroups were adjusted for age, race, education level, marital status, the family poverty income ratio (PIR), body mass index (BMI), hypertension, diabetes, alcohol intake, and smoking status., except the stratification factor itself.

Subgroup analyses and interaction tests were conducted to ascertain the consistency of the relationship between screen-based SB and ED across the general population and to identify any potential variations in different population settings. As illustrated in Fig. 2, the independent positive correlation between screen-based SB and ED remained consistent across all categories, including age, race, education level, marital status, PIR, BMI, hypertension, diabetes, alcohol consumption, and smoking (all p-values for interaction > 0.05). However, the correlation was not statistically significant in the subgroups of individuals under 50 years of age, other Hispanic and other racial groups, those with less than a high school education, a PIR of less than 1.3, and individuals with diabetes, as the 95% confidence intervals included 1.00.

## Discussion

This extensive cross-sectional study, involving 4047 participants, utilized the NHANES) database to explore the relationship between ED and the duration of screen-based SB among adult men in the United States. A strong correlation was found between a higher prevalence of ED and inactive periods exceeding two hours per day, even after adjusting for relevant skewed factors. Furthermore, a noticeable link remained evident after subgroup analysis. These subgroup studies demonstrated that the positive correlation persisted, and none of the stratification variables impacted the stability of the relationship between screen-based SB and ED.

Prior research has explored the correlation between screen-based SB and ED, yet the findings have not reached a consensus. A cross-sectional analysis of data from a prospective cohort study involving health professionals identified a strong correlation between television viewing time and ED in men over 50, even after adjusting for physical activity and other health-related behaviors^27^. A logistic regression analysis of 3941 adult males indicated that lower levels of physical activity were independently associated with increased odds of developing ED^28^. Derby et al. deduced that physical activity status was linked to ED, with sedentary men at the highest risk and men who remained active or initiated physical activity at the lowest risk^29^. Conversely, some studies have reached contradictory conclusions. For instance, no correlation was found between ED and a sedentary lifestyle in a sample of Brazilian men aged 18 to 40^18^. A Mendelian randomization study investigating the causal effect of recreational sedentary behavior on the risk of erectile dysfunction found no evidence that leisure television watching or driving increased the likelihood of developing erectile dysfunction^30^. The discrepancies in these results may be attributed to the use of different assessment tools, varied and sometimes biased population sources, low response rates to questionnaires and interviews, and cultural differences in the willingness to discuss^31^.

While earlier studies have examined the relationship between screen-based SB and ED, the underlying mechanism has received less scrutiny. The biopsychosocial process of sexual function necessitates the coordination of neurological, endocrine, vascular, and psychological systems^32^. Dysfunction or loss in any of these components can potentially lead to ED. Normal penile erection relies on the release of nitric oxide (NO) from endothelial cells and sympathetic nerve endings, serving as the primary neurotransmitter involved in penile erection^33^. SB results in an increase in low and oscillatory shear rates in the lower extremity’s ductus arteriosus^34–37^. Both oscillatory shear stress and low shear stress enhance endothelium-derived reactive oxygen species (ROS) and downregulate endothelial nitric oxide synthase (eNOS) expression and NO production^38,39^. Sedentary lifestyles may also elevate oxidative stress in the vascular endothelium, causing an imbalance in the synthesis of vasodilator and vasoconstrictor chemicals, thereby promoting the development of ED^40^. Consistent evidence from prospective studies and high-quality methodological investigations link SB to CVD, type 2 diabetes, metabolic syndrome, obesity, and all-cause mortality^21–23,41–45^.Type 2 diabetes, metabolic syndrome, and obesity are recognized as common risk factors for erectile dysfunction^7,46^. Vaamonde et al. reported that sedentary individuals exhibited lower levels of follicle-stimulating hormone (FSH), luteinizing hormone (LH), and testosterone than physically active individuals^47^. A recent Mendelian randomization study suggested that leisure SB could influence sex hormone levels, potentially leading to the development and progression of ED^30^. This could provide a novel research direction for understanding the pathogenesis of ED induced by SB.

The present study boasts several strengths. To our knowledge, this is the first study to examine the relationship between screen-based SB and ED using the NHANES database. By leveraging this nationally representative dataset, which accounts for various confounding factors, we enhance the generalizability and reliability of our findings. Our study provides strong evidence of the association between screen-based SB and ED, utilizing validated measurement tools and comprehensive analysis in a large, diverse sample, which has significant implications for public health policy and clinical practice. However, as a cross-sectional study, this investigation has several inherent limitations. Most notably, its design captures associations at a single point in time, which precludes the ability to infer causal relationships. Additionally, reliance on self-reported data may introduce recall bias, particularly for variables such as alcohol consumption, smoking history, and screen time duration, which may not have been accurately reported. Furthermore, unmeasured confounding factors—such as underlying psychological conditions (e.g., depression or anxiety), medication use (e.g., antidepressants or antihypertensives), genetic predispositions, and physical activity—may influence both screen-based SB and ED, potentially biasing the observed associations. A particularly important limitation is the omission of physical activity as a covariate in our multivariable regression analysis. Although screen-based SB was the primary exposure of interest, sedentary behavior and physical activity are distinct yet interrelated domains. For instance, individuals may engage in prolonged screen time while still achieving adequate levels of moderate-to-vigorous physical activity (MVPA), thereby offsetting some of the adverse health effects associated with sedentary lifestyles. Conversely, individuals with limited screen time but insufficient physical activity may still be at elevated risk for ED. The absence of physical activity from our models may result in residual confounding, limiting our ability to isolate the independent effect of screen-based SB on ED and potentially leading to an overestimation of the observed association. Another limitation is the age of the data used, which were collected more than two decades ago. This may affect the generalizability of our findings to current populations. While our findings offer valuable insights for the period studied, future research using more recent data is needed to confirm whether these associations hold true today.

Our study reveals a significant association between screen-based SB and ED, with important clinical and public health implications. Although causality cannot be established in this cross-sectional study, our findings suggest that reducing screen-based SB could be a viable strategy to improve erectile function. Encouraging ED patients to minimize sedentary behavior and engage in regular physical activity could complement current pharmacological treatments, potentially enhancing patient outcomes. Additionally, considering that ED is often an early warning sign of CVD, the observed association between screen-based SB and ED may have broader implications for cardiovascular health. Both ED and CVD share common risk factors and underlying pathophysiological mechanisms, and addressing screen-based SB could help reduce the risk of CVD while simultaneously improving erectile function. These findings underscore the importance of incorporating discussions about sedentary behavior into the treatment plans for ED patients, as reducing screen-based SB could not only improve erectile function but also support overall cardiovascular health. From a public health standpoint, promoting lifestyle modifications—such as reducing screen time and encouraging physical activity—could play a pivotal role in preventing both ED and CVD, ultimately leading to better health outcomes at the population level.

## Conclusion

In conclusion, the findings of this comprehensive cross-sectional study illustrate a robust correlation between screen-based SB and ED among US adults. Subsequent subgroup analysis further substantiated a significant association between the two. However, further research is imperative to validate and replicate our findings, and to elucidate the specific underlying mechanisms.

## Data Availability

Publicly available datasets were analyzed in this study. These data can be found at: www.cdc.gov/nchs/nhanes/.
